# Spatiotemporal gait parameter fluctuations in older adults affected by mild cognitive impairment: comparisons among three cognitive dual-task tests

**DOI:** 10.1186/s12877-023-04281-7

**Published:** 2023-09-27

**Authors:** Shan Du, Xiaojuan Ma, Jiachen Wang, Yan Mi, Jie Zhang, Chengxue Du, Xiaobo Li, Huihui Tan, Chen Liang, Tian Yang, Wenzhen Shi, Gejuan Zhang, Ye Tian

**Affiliations:** 1 Department of Neurology, the Affiliated Hospital of Northwest University, Xi’an No.3 Hospital, Shaanxi, Xi’an, 710018 China; 2 Clinical Medical Research Center, the Affiliated Hospital of Northwest University, Xi’an No.3 Hospital, Shaanxi, Xi’an, 710018 China; 3 Xi’an Key Laboratory of Cardiovascular and Cerebrovascular Diseases, the Affiliated Hospital of Northwest University, Xi’an No.3 Hospital, Shaanxi, Xi’an, 710018 China

**Keywords:** Mild cognitive impairment, Dual tasks, Gait analysis, Spatiotemporal gait parameters, Aging adults, Wearable sensors

## Abstract

**Backgrounds:**

Gait disorder is associated with cognitive functional impairment, and this disturbance is more pronouncedly when performing additional cognitive tasks. Our study aimed to characterize gait disorders in mild cognitive impairment (MCI) under three dual tasks and determine the association between gait performance and cognitive function.

**Methods:**

A total of 260 participants were enrolled in this cross-sectional study and divided into MCI and cognitively normal control. Spatiotemporal and kinematic gait parameters (31 items) in single task and three dual tasks (serial 100-7, naming animals and words recall) were measured using a wearable sensor. Baseline characteristics of the two groups were balanced using propensity score matching. Important gait features were filtered using random forest method and LASSO regression and further described using logistic analysis.

**Results:**

After matching, 106 participants with MCI and 106 normal controls were recruited. Top 5 gait features in random forest and 4 ~ 6 important features in LASSO regression were selected. Robust variables associating with cognitive function were temporal gait parameters. Participants with MCI exhibited decreased swing time and terminal swing, increased mid stance and variability of stride length compared with normal control. Subjects walked slower when performing an extra dual cognitive task. In the three dual tasks, words recall test exhibited more pronounced impact on gait regularity, velocity, and dual task cost than the other two cognitive tests.

**Conclusion:**

Gait assessment under dual task conditions, particularly in words recall test, using portable sensors could be useful as a complementary strategy for early detection of MCI.

## Introduction

Mild cognitive impairment (MCI) is an intermediate transitional stage between normal aging and dementia that is predominately manifested by the decline of cognitive functions involving memory, executive function, language, attention, and visuospatial roles, but not significantly affecting the ability of daily life or meeting the diagnostic criteria of dementia. Epidemiological evidence showed that MCI affected about 20% of the aging individuals worldwide and 10%~15% of them progressed into dementia annually, which in turn leading to decreased quality of life of the population and escalated social economic burden [1–3]. Therefore, early identification and prophylactic intervention of MCI will retard its progression and reduce the transitional risk to dementia in the scenario that there is a lack of effective therapeutic options for reversing dementia. Currently, neuropsychiatric scales depending screen of MCI is time-consuming and requires a professional clinician; the result scores are always influenced by educational level and other personal factors of the subjects [4].

Walking is a common and essential activity orchestrating the functionalities of a variety of muscles and tendons as well as nervous system, which allows people to keep their balance, maintain stability, and move their body from one place to another [5]. Gait refers to the posture of a healthy person when walking and it varies with age, body condition, training and certain disease conditions [6]. Qualitative and quantitative gait assessment using observational scales and instrumental analysis are helpful for the judgement of a normal or pathological movement pattern; it has been used in the clinical diagnosis of orthopedics, rehabilitation, neurological and other diseases, especially in the neurological diseases [7–9]. As a compelling instrument for quantitative gait analysis, the recently applied wearable sensor enables the quantitative gait measurement even to monitor subtle changes during walking, showing great promise in replacing the human observational scale or optical motion capture system due to its low cost, real-time, portable, versatile and informative features [10].

Up to date, multiple lines of studies have confirmed that walking is a learned behavior rather than an automatic process that requires the coordination of cognitive function [11, 12]. Gait performance is associated with individual capacities of executive function and working memory, especially in the dual-task tests [13–15]. Dual-task gait test is a measuring modality to monitor changes of gait parameters when simultaneously performing an attention-demanding cognitive task when walking. Previous studies revealed that the gait patterns in performing dual task tests are disturbed more frequently and pronouncedly in subjects with MCI [16, 17]. Thus, gait analysis in dual task may represent a promising option for the diagnosis and outcome prediction in the target population. However, no consensus has been achieved with regarding to the gait characteristics in distinguishing MCI from normal aging populations due to the varieties in gait measuring tools, loaded cognitive tasks, reported gait parameters as well as individual cognitive capability [18]. Our study aimed to analyze gait parameters (spatiotemporal and kinematic characteristics) of MCI using a wearable sensor under single task and three different cognitive tasks, including counting backwards by 7s, naming animals and words recall. Then we selected important gait features contributing to MCI and compared the performance of gait in different tasks, which may provide objective evidence for the clinical screening of MCI.

## Methods

### Participants

Participants reporting subjective cognitive decline attended the memory clinics of our hospital from December 2020 to December 2021. Eligible subjects met the inclusion and exclusion criteria were enrolled in this cross-sectional study for further analysis. The inclusion criteria were: (1) aging over 50; (2) voluntary to participate in the gait test and provide the informed consent; (3) being able to complete the questionnaire and test independently or with the assistance of the examiners. The exclusion criteria were: (1) patients diagnosed with Alzheimer’s Disease (AD) or dementia; (2) severe verbal, hearing, optical impairments affecting the gait performance; (3) walking difficulty due to lower limbs musculoskeletal diseases, including trauma, arthritis, pain or orthopedic surgeries; (4) central or peripheral nervous diseases that cause gait disorders, such as cerebral stroke, Parkinson’s disease, Lewy body dementia, spinal diseases, normal pressure hydrocephalus; (5) severe concomitant intracranial diseases such as encephalitis, meningitis, seizure, and brain tumors.

Clinical characteristics of the eligible participants were recorded, including age, gender, educational years, smoking or alcohol consuming and medical history of diseases as well as memory complaints. Participants were interviewed about whether they had a subjective memory decline for 3 months or not, and whether they were worried about this decline. Meanwhile, their family history of AD/dementia were asked by “Did your parents ever suffer from Alzheimer’s disease/dementia?”. Sleep disorder referred to reduced sleep duration compared to age and sex matched population, difficulty in falling asleep, frequent awakenings, early morning awakenings, and excessive daytime sleepiness, and was reported by the subjects themselves. Afterwards, the subjects were examined with cognitive scales, daily functioning as well gait performance. The diagnosis of MCI was based on the Petersen criteria [19], briefly as followings, (1) subjective complaints of memory decline or other cognitive decline on self- or informant report or doctors’ assessment; (2) one or more cognitive domain impairment based on neuropsychological testing standardized by the patient’s age and educational background; (3) intact daily functioning in ADL scales and being independent in daily living; (4) not meeting criteria of dementia with a clinical dementia rating (CDR) score less than 0.5. MCI was diagnosed by two independent neurological doctors after comprehensive consideration of clinical characteristics, medical history, daily life ability and neuropsychological testing performance. Subjects didn’t meet the diagnostic criteria were categorized to be cognitive normal control (NC). Finally, 134 patients with MCI and 126 NC were included. This study was approved by the ethnic committee of Xi’an No.3 Hospital and followed the principles of the Declaration of Helsinki. The informed consents were obtained from all participants.

### Cognitive functional assessment

Cognitive function was assessed using the mini-mental status examination (MMSE), Montreal cognitive assessment (MoCA) and CDR scales. Daily functioning was evaluated using Activity of Daily Living (ADL) scale. MMSE scale assesses cognitive functions of 5 domains, including orientation, memory-recall, attention-calculation and language; while MoCA scale further measures visuospatial-executive and abstraction abilities. Both MMSE and MoCA have a total score of 30 points, and a higher score indicates better cognitive ability. One more point will be added to the original total score of MoCA in case of the educational years one attains are less than 12, and the final score < 26 indicates cognitive impairment [20, 21]. MMSE ≤ 24 was used to exclude subjects with possible dementia [22, 23]. CDR scale was used to grade the severity of dementia by scoring from 0 to 3 with score ≥ 1 diagnosed as mild to severe dementia [24]. ADL scale is used to assess daily life ability to assist the diagnosis of MCI [25]. The total score of ADL is 100 and a higher score indicates a more independent status of the participant.

#### Gait assessment

Intelligent device for energy expenditure and activity (IDEEA version 3.0, MiniSun, CA, USA) is an integrated portable system used to monitor energy consumption and physical activities with an accuracy > 98% [26]. The device carries five miniaturized sensors (each 16 × 14 × 4 mm) that are positioned one on the sternum, one on the anterior part of each thigh and one under the plantar arch of each foot, all fixed to the skin by an adhesive tape before the measurement. The sensors measure walking parameters with a data acquisition frequency of 32 Hz and then they project these real-time anthropometric signals of relative position and acceleration to three microprocessors (one advanced microprocessor and two secondary microprocessors) via connected flexible wires. The 32-bit, 32 MHz advanced microprocessor with 200 MB of data storage is attached to the subject’s belt and receives signal input from the sensors positioned on sternum or thighs, and the two secondary microprocessors receive signals from sensors on two feet and then return these data to the advanced microprocessor wirelessly. Then, acquired data are transferred to a peripheral computer via a 12-bit AC/DC converter for signal analysis using an *Actview™* software, which automatically processes 2 h of acquired data in less than 30 s.

IDEEA3.0 system was applied to monitor gait parameters of the participants when performing a single task or three dual-tasks in our study. After checking the device, the sensors and recorders were fixed as the instruction. An error message would appear if the sensors were not in line or the heel of a shoe was higher than 2.5 cm. Then, the subject was asked to walk naturally to acclimate to the device. During this period, green LED lights on the 3 microprocessors (one each) flashing twice per second indicated that all components of the device were correctly connected and worked well. Otherwise, the device was checked again until the alarm signals terminated. The gait test was performed in a quiet room, and the subject was requested to walk 12 m in a straight line on the ground marked with starting and ending points. The gait data of the middle 10 m (automatically selected by the device approximately from the 2nd to 11th meters) were collected for final data analysis. Ensure all gait data from the 5 sensors were collected under four tasks, or this subject would be excluded for further analysis. Gait parameters encompass spatiotemporal (step and stride length, velocity, cadence, stance, swing and double support duration, etc.) and kinematic characteristics (thigh twitch acceleration, thigh swing work, ground reaction forces and heel angle to the ground). Variability of stride time or stride length (stride time/length CV) were calculated by dividing the standard variability of stride time/length to their mean values. Dual task cost (DTC) of velocity was used to assess the influence of cognitive task challenge on gait performance; it was calculated as [(velocity of single task-velocity of dual task)/velocity of single task] × 100%.A total of four gait trials were administered to each patient. Single task trail was performed when walking as usual to gather baseline gait parameters in the absence of cognitive load. For the dual task tests, the subject was asked to finish the three additional cognitive tasks when walking, including serial 100-7, naming animals and specific word recalling, respectively. No priority was allowed to finish either motor or cognitive task. Only one trial was allowed for each task to balance and minimize the effects of learning and fatigue. In order to avoid the events of “walking without thinking” and “thinking without walking” during the gait test, the subject was immediately reminded of “keep thinking” when he/she discontinued the cognitive task, or “keep walking” when he/she stopped walking. In addition, if the subject made an error in the calculation/recall/naming process, the cognitive task proceeded regardless of the error.

### Data analysis

All data were analyzed using R software version 4.1.2 (Vienna, Austria) and SPSS 26.0 (IBM Corp., Chicago, Illinois, USA). The MCI and NC groups were 1:1 matched using propensity scores with a caliper value of 0.4. The matched covariates included gender, age, height, weight, and educational years. Countable data were presented as frequency and percentage (%) and the difference between two groups was compared using Chi-square test or Fisher’s exact test. Quantitative data were expressed as means ± standard deviation (SD) and statistical difference between two groups was analyzed using independent t-test. Difference among multiple groups were compared using one-way analysis of variance (ANOVA) followed by Bonferroni post-hoc test. Important features of gait parameters associating with MCI were selected using recursive feature elimination (RFE) based random forest method and LASSO regression to screen robust variables using a random training set/test set at 7:3. A total of 31 items of gait features were input into Random Forest classifiers with ntree = 500 and mtry = 2 using ‘randomForest’ package of R software. The mean reduction in Gini index, indicated by the principle of impurity reduction, was used to access the feature importance in classification. A greater decrease in Gini index means a higher importance of the feature. LASSO regression with 10-fold cross-validation was carried out using ‘glmnet’ package of R software to centralize and standardize the included variables, and the best-fit lambda (λ) value at λ_min_ or λ_1SE_ was obtained. Univariate and adjusted logistic regression analysis were applied to quantitatively describe the association between selected gait parameters with MCI. *P* < 0.05 was considered statistically significant.

Sample size was calculated using PASS15.0 software (NCSS LLC., Kaysville, Utah, USA)based on a statistical power of 0.8 and a two-tailed α level of 0.05 using a two-sample independent t-test. With reference to the previous studies on means and SD values of gait parameters including velocity and DTC for NC and MCI [1, 27, 28], a minimum of 83 participants per group were required. Power analysis was performed using PASS15.0 software based on a two-sample t-test of the means and SD of gait parameters (such as velocity, DTC, swing time) under the four tasks and the sample size in this study. The significance level was set at 0.05 (two-sided). The estimated power of this study was above 0.813.

## Results

### Baseline characteristics of the participants

**Fig. 1:**
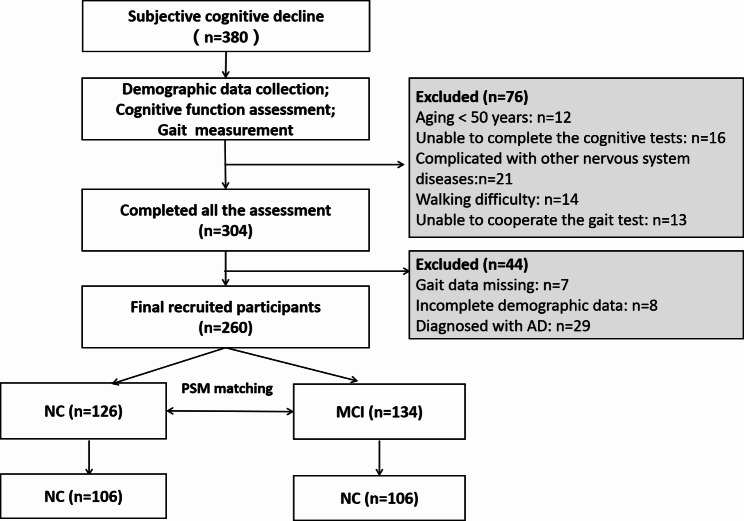
Flowchart of participants included for analysis. PSM: propensity score matching

A total of 380 participants attended the screening of this cross-sectional study, 76 subjects were excluded for not meeting the inclusion and exclusion criteria; 44 subjects were excluded for incomplete demographic data, gait testing data or being diagnosed with AD (Fig. 1). Thus, 260 eligible subjects were enrolled and categorized into cognitive normal control (n = 126) and MCI (n = 134) based on the diagnostic criteria. The baseline clinical characteristics was shown in Table 1. The data showed that in the overall population, the participants with MCI had higher body weight as well as BMI, and lower educational level with 6–9 years accounting for 42.5% of the group. The two groups had similar comorbid conditions such as cardiovascular diseases, subjective sleep disorder and family history of AD. The MoCA sores were 26.72 ± 1.98 in NC group and 21.52 ± 2.35 in MCI group, indicating a significant difference between the two groups (*P* < 0.05). Meantime, there was statistical difference in MMSE scores between them (*P* < 0.001). To balance the significant confounding variables potentially affecting cognitive and gait functions, a matched population at a ratio of 1:1 in NC and MCI group was created using propensity score matching (PSM) method after adjusting gender, age, height, weight, and educational years. Eventually, a total of 212 subjects, 106 in each group, were matched and most of the significant imbalances were removed without yielding other imbalanced variables between the two groups. As expected, significant difference on MMSE and MoCA remained existing between the NC and MCI groups after PSM matching (*P* < 0.001 for each comparison).

**Table 1:** Baseline characteristics of the overall and matched populations

Characteristics	Overall Population			Propensity Score-Matched (1:1) Population ^a^		
	NC (n = 126)	MCI (n = 134)	*P* ^*b*^	NC(n = 106)	MCI(n = 106)	*P* ^*b*^
Age (years)			0.122			0.319
50 ~ 59	55 (43.7)	42(31.3)		44(41.5)	33(31.4)	
60 ~ 69	55 (43.7)	70(52.2)		47(44.3)	56(53.3)	
70 ~ 79	16 (12.7)	20 (14.9)		15(14.2)	15(14.3)	
≥ 80	0 (0.0)	2 (1.5)		0(0.0)	1(1.0)	
Female n (%)	89 (70.6)	74 (55.2)	0.010	69(65.1)	65(61.3)	0.669
Height (cm)	163.08 ± 7.33	163.52 ± 8.11	0.647	163.56 ± 7.48	162.86 ± 7.51	0.499
Body weight (kg)	61.46 ± 9.84	65.52 ± 10.83	0.002	63.02 ± 9.95	63.96 ± 10.47	0.502
BMI (kg/m^2^)	23.16 ± 2.87	24.32 ± 2.97	0.002	23.54 ± 2.86	24.04 ± 3.06	0.210
Education n (%)			< 0.001			0.068
< 6 years	12 (9.5)	13 (9.7)		12(11.3)	10(9.4)	
6–9 years	25 (19.8)	57 (42.5)		25(23.6)	43(40.6)	
9–12 years	53 (42.1)	46(34.3)		50(47.2)	37(34.9)	
> 12 years	36 (28.6)	18 (13.4)		19(17.9)	16(15.1)	
Martial status (%)			0.442			0.318
Single	0(0.0)	1(0.8)		0(0.0)	1(0.9)	
Married	121 (96.0)	122 (91.0)		102(96.2)	95(89.6)	
Divorced	1(0.8)	2(1.5)		1(0.9)	2(1.9)	
Widowed	4(3.2)	9(6.7)		3(2.8)	8(7.5)	
Living alone n (%)	9 (7.1)	15(11.2)	0.259	7(6.6)	14(13.2)	0.167
Exercise n (%)			0.156			0.149
Never	8(6.3)	6(4.5)		6(5.7)	5(4.7)	
< 3 times/week	49(38.9)	39(29.1)		43(40.6)	30(28.3)	
≥ 3 times/week	69(54.8)	89(66.4)		57(53.8)	71(60.7)	
Smoking n (%)	31 (24.6)	48 (35.8)	0.075	31(29.2)	32(30.2)	0.358
Alcohol use n (%)	29 (23)	40 (29.9)	0.326	28(26.4)	27(25.5)	0.809
Hypertension n (%)	46(53.6)	57(42.5)	0.321	44(41.5)	44(41.5)	1.000
Diabetes n (%)	11 (8.7)	20 (14.9)	0.123	9(8.5)	17(16.0)	0.094
Hyperlipemia n (%)	38 (30.2)	41(30.6)	0.939	33(31.3)	31(29.2)	0.881
Coronary arterial disease (%)	20 (15.9)	18(13.4)	0.578	17(16.0)	14(13.2)	0.698
Sleep disorder n (%)	67 (53.2)	65 (48.5)	0.452	59(55.7)	54(50.9)	0.582
Family history n (%)	14 (11.1)	10 (7.5)	0.310	10(9.4)	10(9.4)	1.000
MMSE	28.91 ± 1.17	27.35 ± 1.65	< 0.001	28.90 ± 1.05	27.30 ± 1.64	< 0.001
MoCA	26.72 ± 1.98	21.52 ± 2.35	< 0.001	26.22 ± 2.39	21.01 ± 2.52	< 0.001

Note: ^a^ The MCI and NC groups were 1:1 matched using propensity scores with a caliper value of 0.4 based on the covariates of gender, age, height, weight, and educational years

^b^*P*-value between NC and MCI groups in the overall population or propensity score-matched population was determined using chi-square or Fisher’s exact test for categorical variables presented as n (%) or using independent t-test for continuous variables presented as means ± SD.

### Important feature selection of gait parameters associating with cognitive function

**Fig. 2:**
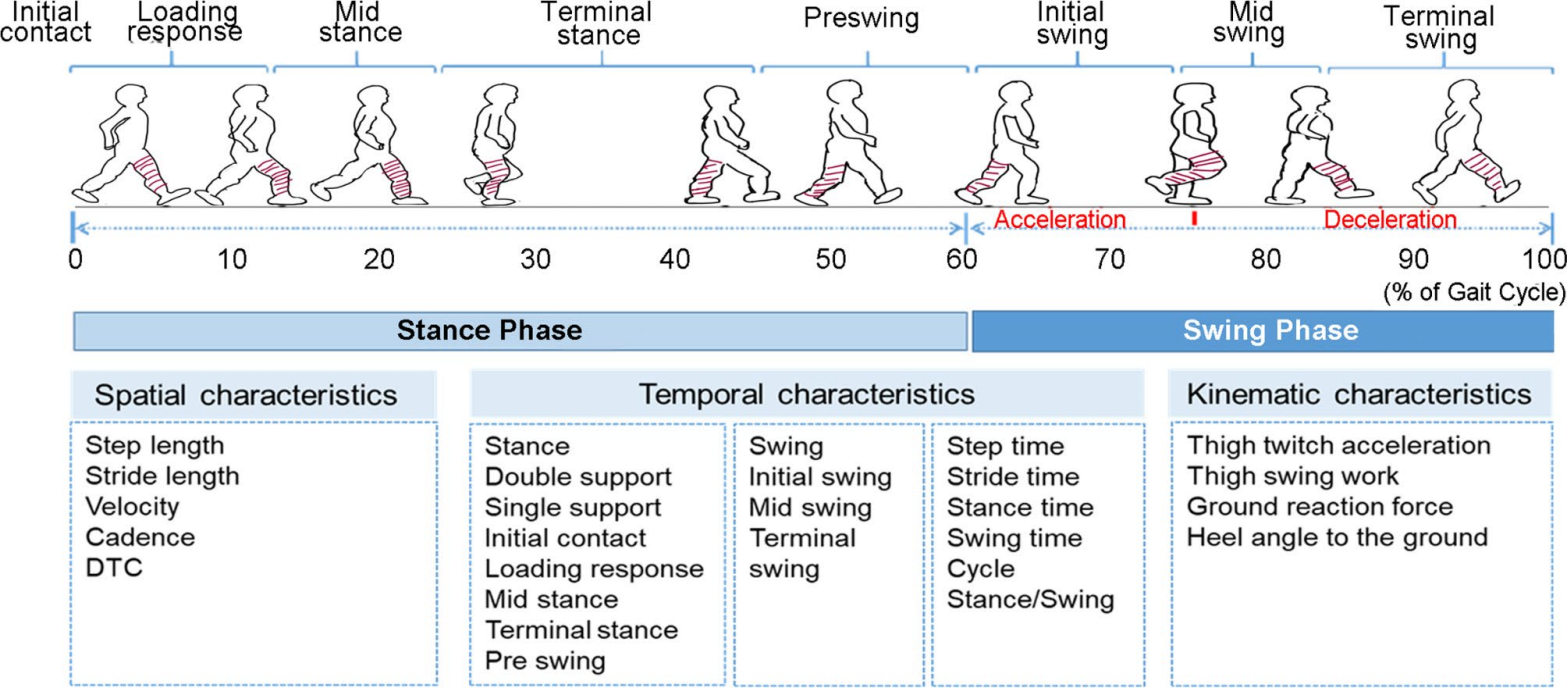
Schematic diagram of functional phases of a normal gait cycle and the measured spatiotemporal parameters using a wearable gait measuring device (IDEEA 3.0 system)

In this study, a panel of spatiotemporal and kinematic gait parameters as listed in Fig. 2 was measured using portable sensors in all the enrolled participants. To analyze the association between gait performance and cognitive function in the matched population, all gait parameters (31 gait characteristics) were pooled into a random forest and a LASSO regression model to select important features contributing to MCI in single task and three dual-task modes. As shown in Table 2, in different task modes, top 5 important features based on random forest model were selected and 5 ~ 6 significant features at λ_min_ in LASSO model were shown. For the single task, the common ranked features included variability of stride length and thigh twitch acceleration. While, in double task of serial 100-7 test and naming animals test, DTC and gait cycle of mid stance and terminal swing were common features associated with MCI. In words recall test, only DTC was selected as common feature by the two algorithms. Therefore, selection preference could be observed in different models, and the combined features were further analyzed in each task.

**Table 2:** Important gait characteristics selection associated with cognitive function in matched populations

Tasks	Random forest		LASSO regression		The combined features
	**Features**	**Importance**	**Features**	**Importance**	
Single task	Stride length CV	0.577	Thigh swing work	-0.120	Stride length CV Stride time CV Load response Initial swing Mid swing Thigh twitch acceleration Thigh swing work
	Mid swing	0.555	Load response	-0.012	
	Thigh twitch acceleration	0.531	Stride length CV	0.010	
	Stride time CV	0.524	Thigh twitch acceleration	0.001	
	Initial swing	0.511			
Subsequent 100-7	Mid stance	0.592	Swing time	-0.484	Stance time CV Swing time Terminal swing Mid stance Thigh swing work DTC
	Thigh swing work	0.547	Terminal swing	-0.032	
	Stance time CV	0.540	Mid stance	0.002	
	DTC	0.534	DTC	0.002	
	Terminal swing	0.528			
Naming Animals	Terminal swing	0.565	Stride length	-0.271	Stride length Stride length CV Terminal swing Mid stance DTC Stride time CV Swing time Swing time CV
	Stride time CV	0.533	Swing time	-0.225	
	Swing time CV	0.528	Terminal swing	-0.039	
	DTC	0.526	Mid stance	0.013	
	Mid stance	0.526	Stride length CV	0.008	
			DTC	0.003	
Words recall	Stride time CV	0.568	Swing time	-0.117	Velocity Stride length Stride time CV Swing time DTC Mid stance Terminal swing Thigh swing work Thigh twitch acceleration
	Thigh swing work	0.543	Terminal swing	-0.015	
	Velocity	0.540	Thigh twitch acceleration	-0.021	
	DTC	0.532	DTC	0.002	
	Stride length	0.522	Mid stance	0.001	

Note: CV: coefficient of variation, calculated by dividing the standard variability to mean value. DTC: dual task cost of velocity, calculated as [(velocity of single task-velocity of dual task)/velocity of single task] × 100%. Top -ranked features associating with MCI were selected using recursive feature elimination (RFE) based random forest (randomForest package) and LASSO regression (glmnet package) algorithms of R-version-4.1.2

### Logistic regression analysis of the selected gait parameters associating with MCI

**Fig. 3:**
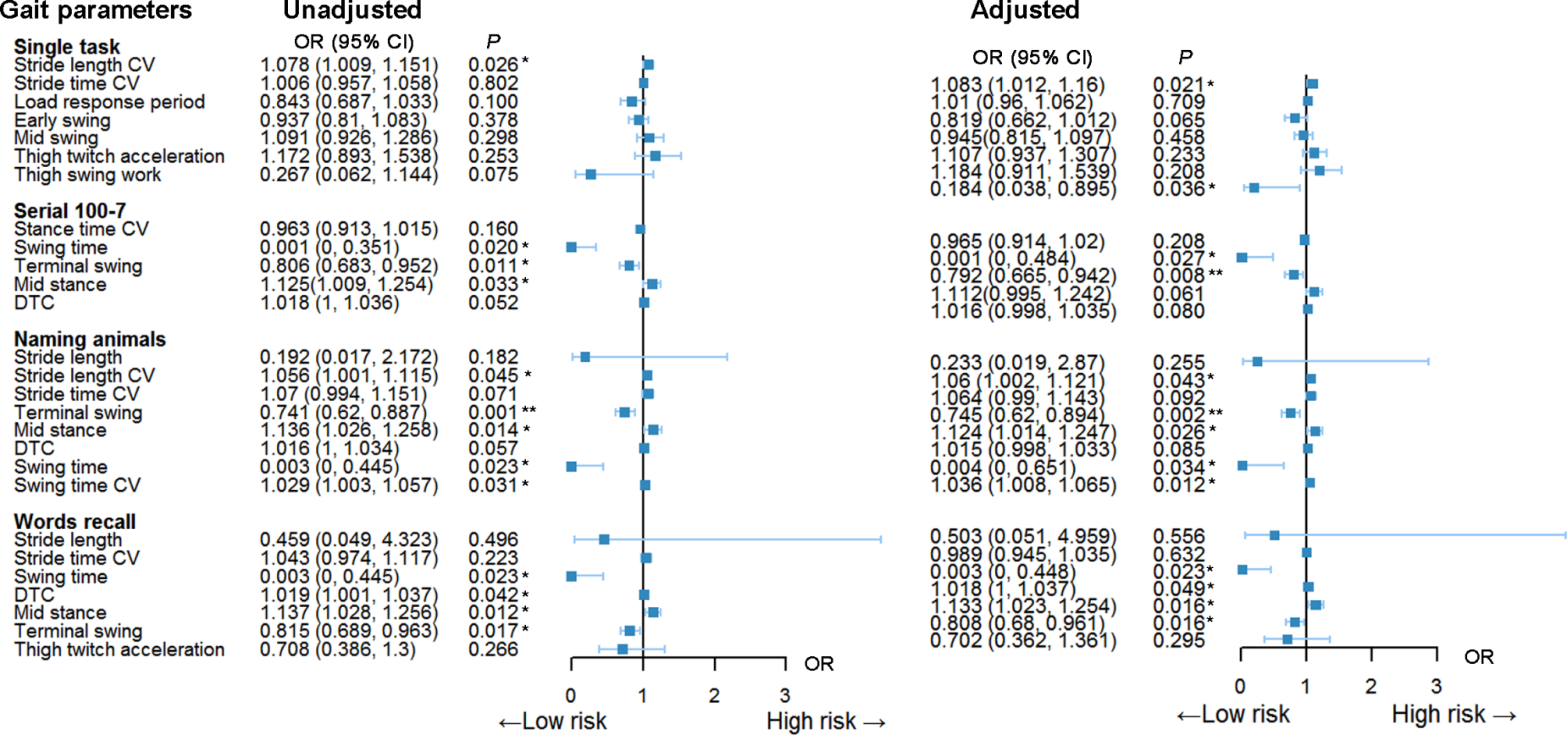
Forest plots of logistic regression models of gait parameters in different task condition associating with MCI. The left panel of forest plot presents the unadjusted univariate logistic model using dichotomous NC/MCI as dependent variable and the left panel shows the multivariate logistic model predicting MCI after adjusting for age, gender, height, weight, and educational level. OR with 95% CI and *P* values were estimated using logistic regression. The square represents the OR value and the connected whiskers show corresponding 95% CI. ^*^P < 0.05 and ^**^P < 0.01 indicate statistically significant estimates

To further delineate the association between the above selected important gait features and MCI, an univariate logistic model and a multivariate logistic model adjusting gender, age, height, body weight were created, and the odds ratio (OR) with 95% confidence interval (CI) and testing significance were shown in a forest plot in Fig. 3. In the single task, only stride length CV was identified as a risk factor for MCI in the adjusted logistic model (OR = 1.083, 95% CI: 1.012–1.060, *P* = 0.021). In serial 100-7 dual task, swing time and terminal swing phase were screened as two independent risk factors for MCI, with shorter swing period and lower terminal swing cycle indicating higher risk of MCI (OR = 0.001, 95% CI: 0-0.484 and OR = 0.792, 95% CI: 0.665–0.942). In dual task of naming animals, in addition to the above variables, mid stance phase, stride length CV and swing time were also contributing factors indicating MCI. When performing a word recall test, DTC, swing time, mid stance, and terminal swing independently predicted cognitive impairment. Collectively, the logistic regression analysis suggested that mid stance, terminal swing, and swing time were filtered as common hallmarks of gait predicting cognitive decline.

### Comparisons of gait parameters between normal aging people and cognitively impaired population

**Fig. 4:**
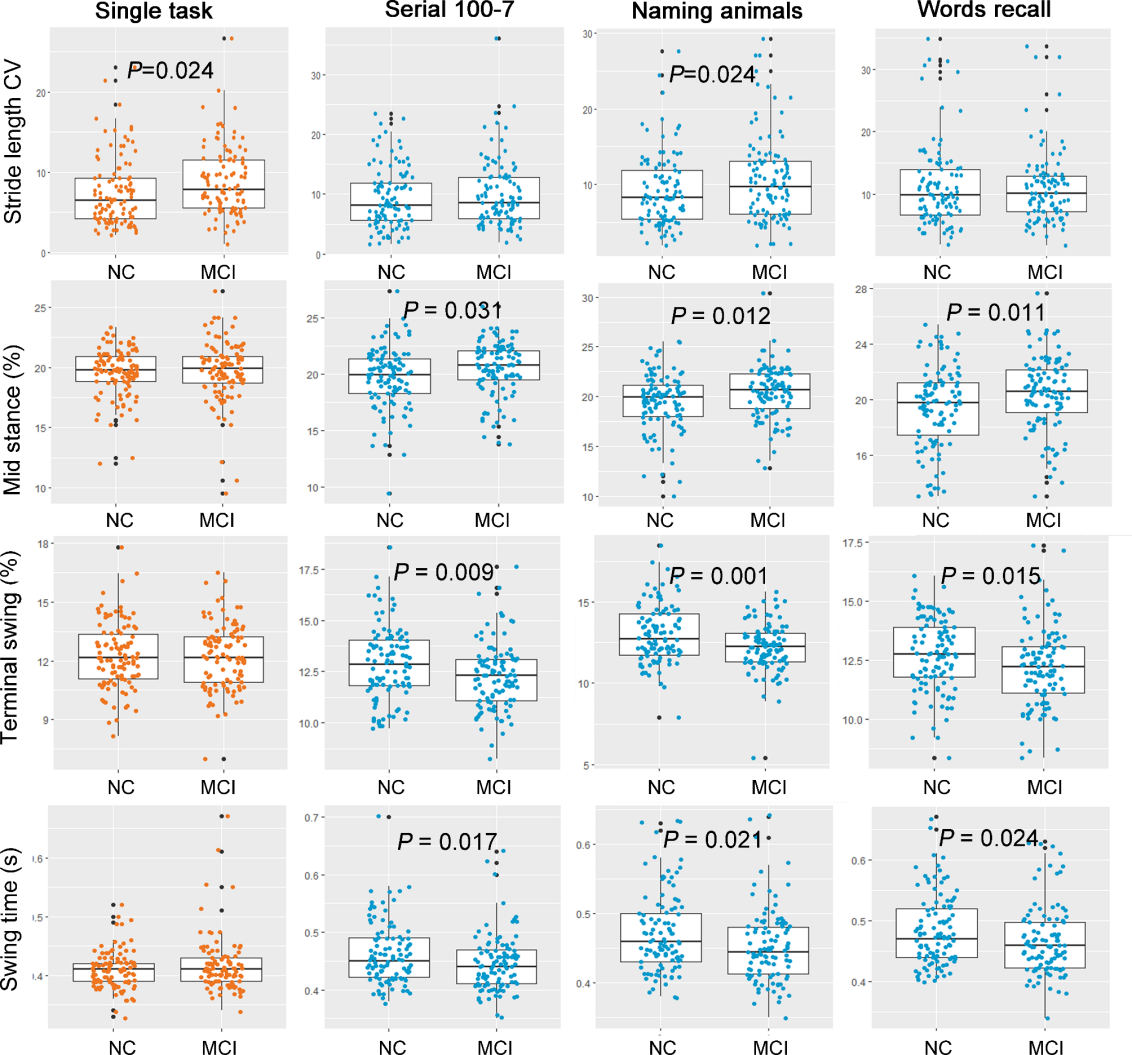
Scattering box plots of gait parameters in NC and MCI participants under different tasks. The four vertical plots in each column show various gait parameters measured under a certain task, while four horizontal plots present a certain gait parameter under four tasks. The jitter dots indicate the absolute gait value of each subject in NC or MCI group. The center line of the box plot indicates the median value and the bounds of box indicate first quartile (Q1) and the third quartile (Q3) values in each group. Interquartile range (IQR) refers the difference between Q3 and Q1. The connected whiskers in the box plot indicate values within the range of the upper limit (Q3 + 1.5×IQR) and lower limit (Q1-1.5×IQR). The *P* values were compared between NC and MCI groups and analyzed using independent t-test

Based on the above results, we further traced and compared the changes of these filtered gait parameters in the two groups. As shown in the scatter-box plots in Fig. 4, stride length CV obviously increased in MCI subjects in the condition of single task and naming animals dual task test compared with NC group (*P* = 0.024 for each comparison). Although elevated stride length CV was also found in MCI under the dual tasks of serial 100-7 and words recall, it didn’t reach statistical significance. Mid stance and terminal swing phase are two temporal gait parameters accounting for approximately 18% and 11% of the whole gait cycle for normal adults, respectively. In our study, the mean mid stance of NC group was 19.59% in performing single task, which was not differed from MCI group of 19.73 ± 2.51% (*P* = 0.066). In the three dual tasks, the mean mid stance (%) of MCI group increased to 20.46% in serial100-7 test, 20.39% in naming animals test and 20.42% in words recall test, which were all significantly higher than that in NC group. For the terminal swing, the two groups had comparable percentage under single task condition; reduced terminal swing was found in MCI group than NC group when performing dual tasks. Likewise, there was no significant difference in the mean swing time between NC and MCI in single task; however, MCI group exhibited significantly decreased swing time than NC when performing three cognitive tasks.

### Effects of different dual cognitive tasks on the performance of gait test

**Fig. 5:**
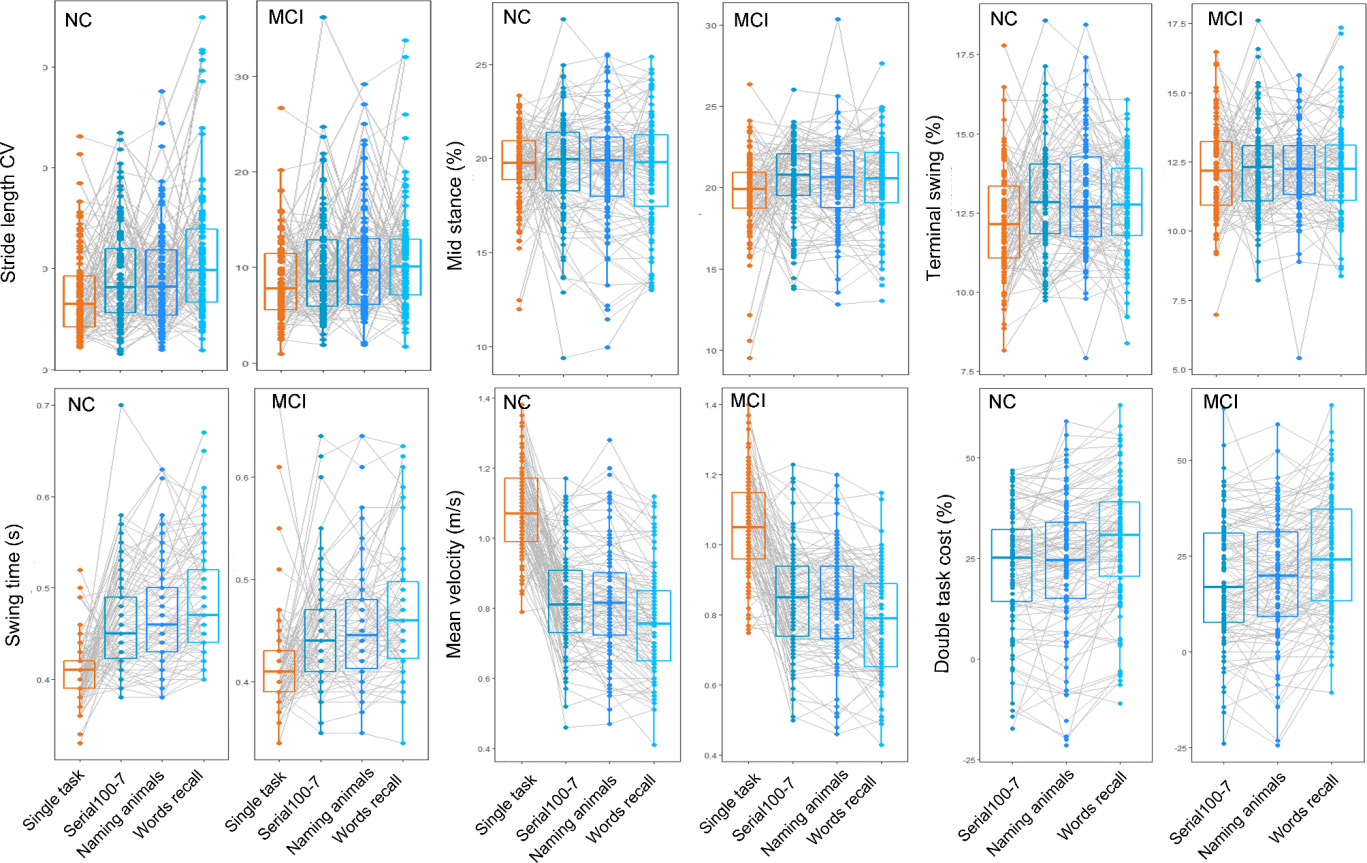
Comparisons of gait performance under single task and three different dual tasks. Six gait parameters in performing the four tasks were separately compared in NC (left view) or MCI (right view) groups. The jitter dots indicate the absolute gait value of each subject in NC or MCI group. The box plot show the Q1, median, and Q3 values in each group. The connected whiskers in the box plot indicate the upper limit and lower limit. The gray lines connecting two dots trace the changes of each participant’s gait parameter under various tasks. Difference among multiple tasks were compared using ANOVA followed by Bonferroni post-hoc test

To elucidate the effect of different cognitive tasks on the performance of gait test, we compared the above screened gait parameters, velocity and DTC between single task and three dual tasks as illustrated in Fig. 5. The ANOVA analysis revealed that there was statistical difference in the stride length CV among the different tasks both in NC group and MCI group (F = 8.819, *P* < 0.001 and F = 3.225, *P* = 0.026), and subjects tended to have higher stride length CV when performing the dual task gait tests, particularly in finishing the words recall test. The percentage of mid stance didn’t differ significantly under the four modes of tasks either in NC or MCI group. Statistical difference on percentage of terminal swing phase was shown among the four task modes in NC group (F = 3.96, *P* = 0.008) with significantly higher values in counting backward by 7 test and enumerating different animals. Whereas, this difference was not shown in MCI group. Besides, participants had higher expenditure in swing time when performing the dual tasks either in NC or MCI groups (F = 44.11, *P* < 0.001 and F = 18.32, *P* < 0.001, respectively), with a stepwise increase between serial 100-7 and naming animals, especially higher in words recall test than the other two dual tasks (both *P* < 0.05). Because velocity was an important hallmark parameter of gait, we then evaluated the changes of velocity under different tasks. Notably, slower speed was monitored in dual tasks in both NC and MCI groups, and the mutual comparison following ANOVA suggested much lower velocity in words recall test comparing with counting backward by 7 test and enumerating different animals test (both *P* < 0.05). Likewise, significantly higher DTC on velocity was required when finishing the words recall test than that in other task in both NC and MCI groups.

## Discussion

This study explored the gait performance in normal aging people and subjects with cognitive decline in finishing single or three different dual task paradigms. In a significant confounders balanced population, important variables associating with MCI were filtered using random forest and LASSO models in each task. The commonly differed variables between NC and MCI groups were temporal gait characteristics including swing time, mid stance, and terminal swing as well as spatial characteristic DTC in the dual task modes as further evaluated by logistic regression analysis. Compared with single task, all subjects walked slower when executing an additional cognitive task as reflected by the substantially decreased velocity, and this decrease was particularly obvious in finishing the words recall task requiring the memory capability. Therefore, disturbed temporal gait parameters under dual tasks may provide objective evidence for the clinical screening of cognitive decline in aging population.

In our study, by comparing the demographic data of the 260 enrolled participants categorized as NC and MCI, we found that participants in MCI group had significantly higher body weight as well as BMI, and lower educational levels, indicating that obesity and educational background may associate with cognitive decline. Several epidemiological studies reported that people with higher BMI had a greater risk for developing MCI and AD [29–31]. Obesity was associated with lower brain volumes in cognitively normal elderly subjects and higher BMI was associated with brain volume deficits in both AD and MCI [32]. Educational background is a well acknowledged contributor associating with cognitive status. Multiple lines of evidence revealed that higher education level served as a protective factor to reduce the risk of MCI and AD [33, 34]. Rolstad et al. reported that stable MCI patients with higher education had lower concentrations of t-tau as compared to those with lower education, and higher education may offer protection against tauopathy [35]. Therefore, propensity score matching method was used to balance these significant confounding variables between the two groups. Additionally, although MCI and NC groups present comparable frequency of sleep disorder in this study, it still exerts an adverse influence on individual’s cognitive function. A longitudinal population-based cohort study revealed that sleep disturbance was associated with worse future cognitive performance for the 60-year-olds [36]. A significant V-shaped association is shown between sleep duration and MCI/dementia risk in women with either short (≤ 6 h/night) or long (≥ 8 h/night) sleep duration involving higher risk of cognitive impairment [37]. Sleep abnormalities can accelerate AD pathophysiology, promoting the accumulation of amyloid-β and phosphorylated tau [38]. In our study, about half of the participants in NC or MCI groups reported sleep disorder with regarding to abnormal sleep duration or sleep latency, indicating that sleep patterns underwent significant modifications in micro and macrostructure along with aging. Therefore, sleep problems should be noticed and individualized interventions targeting sleep disturbances in elderly people should be recommended to prevent or decelerate conversion to dementia. Besides, further studies are needed to expand our understanding on the contribution of sleep disorder to cognitive decline and the associated behavior, such as walking and gait characteristics.

Accumulating studies supported that walking is a complicated activity involving both motor and cognitive functions and their interplay or coordination rather than an automatic process [15]. Based on this rational, gait characteristics of patients with cognitive decline have been discussed in several previous studies [16, 39, 40]. A meta-analysis summarizing the effect of MCI on gait involving 11 studies concluded that MCI affected specific gait parameters, and these changes were particularly pronounced when subjects were challenged cognitively [18]. In these retrieved studies, four criteria were used for the diagnosis of MCI and three modalities of instruments were used to measure the gait function, including electronic walkways, force plates and body-worn sensors. Items of gait parameter varied substantially across the included studies even when the same instrument was used, and only routine spatial gait parameters were reported. Therefore, more studies are needed due to insufficient evidence of these heterogeneous studies. In the past decades, wearable sensors were prevalent in gait analysis and proved to be useful as they permitted a simple, objective assessment of human gait [41]. In our study, a wearable sensor was used and about 30 items of gait characteristics were captured. This portable device could collect spatial-temporal and kinetic gait features. These measured features were pooled into algorithms and spatial-temporal features were selected for their association with MCI, indicating their sensitivity in detecting cognitive decline.

We evaluated the gait performance of normal aging people and patients with MCI in single task and three different dual tasks challenging cognitive capabilities. It was found that participants with MCI exhibited significantly higher variability of stride length in single task than the normal controls, suggesting the disturbed gait regularity in these subjects. This gait disturbance also can be reflected under dual task condition when performing naming animals test. The greater variability of stride length in MCI were also reported in other studies [42, 43]. Moreover, our study identified that in dual task mode, temporal gait parameters swing time, in paralleled with the percent of terminal swing phase in a gait cycle greatly reduced in cognitively impaired individuals compared to normal aging people. A gait cycle is defined as the period from the initial contact of one foot to the following occurrence of the same event with the same foot. Currently, the gait cycle could be partitioned at different granularity based on the measuring methods of events and temporal phases. With the advancement of measuring techniques, wearable sensors emerge as the most promising device for extraction and analysis of larger number of features of gait, which enable the gait segment into more sub-phases (ranging from 2 to 8 sub-phases) [10]. Nowadays, the widely used wearable sensors for gait phase recognition include linear accelerometer, gyroscope, force-based measurements, electromyographic sensors, inertial measurement units, and joint angular sensors. It is concluded that analysis of the acceleration allowed researchers to recognize a greater granularity of gait cycles, such as the sub-phases of the swing phase [44]. In our study, IDEEA system carrying five accelerometers were used to monitor body and limb motions constantly. These motion signals are first preprocessed by signal conditioners and then output as electric signals representing motion and speed. Afterwards, the electric signals fed to the microprocessor data acquisition unit at high rate through a cable. Thus, the motion signals were transformed to time-serial waveform curves showing amplitude of relative position and acceleration information of the subject’s gait. Currently, numerous valuable methods are used for gait phase partitioning based on the waveform curves. Basically, threshold method, time-frequency analysis, and peak heuristic algorithms are used for event and phase detection. While, machine learning based approaches, containing various algorithms such as Hidden Markov models, Deep Learning Neural Network, and so on, are becoming mainstay techniques. Different computation methodologies provide different performances regarding the parameters such as the number of detectable phases, events, and detection delay [5]. Taborri et al. summarized that both threshold-based methods and machine-learning approaches could obtain satisfactory performance in gait phase detection and permit the sub-partitioning of the swing phase [44]. In IDEEA system, wavelet-based algorithm and Bayesian analysis are used to analyze the trajectory and recognize the phases of gait. The different combinations of signals from those five sensors represent different physical activities. Thus, the sensors and algorithms applied in this study allow to segment the gait cycle into eight sub-phases, particularly for the swing phase partitioning [44]. Swing time refers to the duration between the Toe-Off and the Heel-Strike of one leg inside a gait cycle, which takes 0.36 ~ 0.40 s and accounts for approximate 40% of this cycle. During this phase, the leg first pushes backwards and then swings forwards, transforming the potential energy into kinetic energy, and resulting in the highest values in the acceleration and angular velocity signal to propel the forward motion of the whole body [10]. The actual swing is divided into three phases: initial, mid and terminal swing phase at approximately 60–75%, 75–85% and 85–100% of the gait cycle, respectively. The terminal swing phase, the ending of swing phase and the entire gait cycle, is responsible for decelerating forward motion of the lower limbs and preparing for foot landing for the next gait cycle. The decreased swing time and terminal swing in MCI may indicate an impaired capacity for moving forwards when conducting a cognitive task. Meanwhile, we found that MCI patients showed elevated mid stance than the control. The mid stance is the only phase of a single support for whole gravity, which functions to maintain the stability of the knee joint and control the forward inertial motion of the limb. Therefore, the participants with MCI may experience insufficient walking stability and posture control when performing a dual task that needed being compensated by prolonged mid stance. Conventionally, gait velocity, cadence and stride length were frequently focused in MCI patients in previous studies, while the detailed temporal characteristics were rarely portrayed [45]. Thus, a comprehensive understanding of specific gait pattern in MCI population is needed since gait is a complex integrated activity and slow speed is a nonspecific variable linking with many subjective and objective factors [46]. We did not observe substantial difference in gait speed between MCI and control under single and dual tasks in spite adjusting the covariates of age, sex and height or weight. The previous study tended to believe that patients with MCI exhibited slower speed under single or dual task conditions although controversy existed in other studies showing no difference between them [47–49]. It can be explained that our study recruited subjects aging over 50 years with 85% of them ranging from 50 to 70, which may differ from other studies in age distribution. Meanwhile, no priority was permitted to perform motor or cognitive tasks, thus, the attention capacity and preference in allocating these resources between the two tasks may also affect the performance in gait speed.

In our study, three different dual cognitive tasks compassing subsequent 100-7, naming animals and words recall were loaded onto the gait assessment, and their impacts on gait performance of the participants were evaluated. Overall, all subjects exhibited significantly slower speed when conducting an additional cognitive task comparing with performing a single task, which was well acknowledged in other studies [45]. Meantime, we noticed that subjects had poorer gait performance in words recall test, as reflected by the more obvious decrease in velocity and higher velocity cost comparing with the other two tests. The arithmetic tasks (subsequent subtraction by 7s from 100) and verbal fluency tasks (enumerating animal names) seemed to have comparable effects on gait function. The three dual tasks challenged different cognitive resources. Serial 100-7 is a mental tracing task engaging numerical processing skills; verbal fluency tasks involving semantic knowledge and retrieval processes, while words recall activities engaging episodic memory encoding and retrieval processes. Activation of neural circuit in bilateral prefrontal cortices are implicated in all these tasks [50]. Related studies showed that verbal fluency tasks resulted in similar magnitude of interference as mental tracking tasks when walking [51]. In our study, words recall trial caused higher burden and disturbance on gait, indicating that this task is more complex and high-demanding, which is consistent with the high frequency of memory impairment in MCI. As depicted by Schwenk et al., more complex cognitive tasks seem to be required to elicit the gait speed differences between healthy from cognitively declined subjects [46].

## Conclusions

Our study demonstrated dual task gait measurement is superior than the single task in discriminating cognitively decline people from normal aging population. In dual task condition, all subjects walked slower than that in single task mode, and the decrease of velocity were particularly obvious in completing the words recall task that competes the memory capability when walking. Commonly, participants with MCI exhibited decreased swing time and terminal swing phase, as well as increased mid stance and variability of stride length comparing with normal aging participants when performing the dual tasks, indicating the insufficient walking stability and posture control in these patients. This study is limited for its cross-sectional nature and a dynamic follow-up is advocated. Besides, multicenter study involving more participants should be performed to validate the conclusion.

## Data Availability

The raw data supporting the conclusions of this article will be made available by the corresponding authors, upon reasonable request.
